# Fine-scale substrate heterogeneity does not affect arthropod communities on green roofs

**DOI:** 10.7717/peerj.6445

**Published:** 2019-03-19

**Authors:** Bracha Y. Schindler, Amiel Vasl, Leon Blaustein, David Gurevich, Gyongyver J. Kadas, Merav Seifan

**Affiliations:** 1 Institute of Evolution and Department of Evolutionary and Environmental Biology, University of Haifa, Haifa, Israel; 2 Environmental Research Group, Sustainability Research Institute, University of East London, London, United Kingdom; 3 Department of Desert Ecology, Swiss Institute for Dryland Environmental and Energy Research, The Jacob Blaustein Institutes for Desert Research, Ben-Gurion University of the Negev, Sede Boker, Israel

**Keywords:** Formicidae, Collembola, Soil heterogeneity, Vegetated roof, Biodiversity

## Abstract

Green roofs, which are roofs with growing substrate and vegetation, can provide habitat for arthropods in cities. Maintaining a diversity of arthropods in an urban environment can enhance the functions they fill, such as pest control and soil development. Theory suggests that the creation of a heterogeneous environment on green roofs would enhance arthropod diversity. Several studies have examined how arthropod diversity can be enhanced on green roofs, and particularly whether substrate properties affect the arthropod community, but a gap remains in identifying the effect of substrate heterogeneity within a green roof on the arthropod community. In this paper, it is hypothesized that creating heterogeneity in the substrate would directly affect the diversity and abundance of some arthropod taxa, and indirectly increase arthropod diversity through increased plant diversity. These hypotheses were tested using green roof plots in four treatments of substrate heterogeneity: (1) homogeneous dispersion; (2) mineral heterogeneity—with increased tuff concentration in subplots; (3) organic heterogeneity—with decreased compost concentrations in subplots; (4) both mineral and organic heterogeneity. Each of the four treatments was replicated twice on each of three roofs (six replicates per treatment) in a Mediterranean region. There was no effect of substrate heterogeneity on arthropod diversity, abundance, or community composition, but there were differences in arthropod communities among roofs. This suggests that the location of a green roof, which can differ in local climatic conditions, can have a strong effect on the composition of the arthropod community. Thus, arthropod diversity may be promoted by building green roofs in a variety of locations throughout a city, even if the roof construction is similar on all roofs.

## Introduction

Green roofs, which are roofs containing growing substrate and planted with vegetation, can provide habitat for a diversity of plant species and arthropod species in an urban setting. Green roofs have been shown to provide habitat for invertebrates in need of conservation (Brenneisen, 2006; Kadas, 2006), and may be as effective in supporting a diversity of arthropods as ground-level habitats (reviewed in Williams, Lundholm & MacIvor, 2014). In general, heterogeneous environments that provide a diversity of niches may promote greater biodiversity (Stein, Gerstner & Kreft, 2014). There are relatively few studies of the effects of heterogeneity in soil on biodiversity (Stein, Gerstner & Kreft, 2014), but this subject should be of interest since soil heterogeneity could theoretically contribute to greater plant diversity (Vasl & Heim, 2016). If substrate heterogeneity results in higher plant diversity, there could be an indirect effect on arthropod diversity; diversity may be higher overall, because of the diversity of niches provided by the plants. However, different functional groups, such as herbivores and predators, may have a negative or positive response to high plant diversity. Heterogeneity in the soil could also contribute directly to diversity of arthropods associated with the soil. For example, diversity of microhabitats in the soil or litter has been shown to have a positive effect on mite diversity (Anderson, 1978; Hansen & Coleman, 1998) and collembolan diversity (Kaneko & Salamanca, 1999). In this study, the collective influence of direct and indirect effects of soil heterogeneity on the entire arthropod community was examined. The green roof setting allowed for controlled manipulation of substrate at a scale relevant for plants and arthropods, as well as contributing to knowledge on the building of biodiverse green roofs.

A positive relationship between plant and arthropod diversity, affecting all functional groups similarly, may occur via diversity-biomass relationships (Borer, Seabloom & Tilman, 2012). According to this theory, plant diversity has a positive effect on plant biomass, which has a positive effect on arthropod biomass (Borer, Seabloom & Tilman, 2012). This effect generally results in a positive effect on arthropod diversity, because higher biomass increases the probability of additional species being present (Borer, Seabloom & Tilman, 2012). An alternative mechanism is that plant diversity might have a direct positive effect on arthropod diversity, which is not the result of an effect on arthropod abundance. This may occur because an increase in plant diversity increases the diversity of resources available to specialist arthropods (Haddad et al., 2009).

While some theories predict an increase in overall arthropod diversity and abundance with increasing plant diversity, others predict differing responses in the abundance and diversity of different trophic groups within the arthropods. The enemies hypothesis proposes that a more complex vegetational habitat, which would result from higher plant diversity, would support a higher diversity of herbivores, resulting in higher predator and parasitoid abundances (Root, 1973). Thus, while predator and parasitoid abundance is expected to increase with plant diversity, herbivore abundance is expected to be lower due to predation on herbivores (Duffy et al., 2007). A complementary hypothesis, the resource concentration hypothesis, predicts that when vegetation diversity is low, there is a high abundance of a few specialist herbivore species, so herbivore diversity is low and abundance is high (Root, 1973). Thus, there would be a low diversity of predators and parasitoids—species that are adapted well to the herbivore and plant species available (Root, 1973). The predictions of these hypotheses are supported by many studies (Siemann et al., 1998; Knops et al., 1999; Koricheva et al., 2000; Haddad et al., 2001; Haddad et al., 2009; Wilsey & Polley, 2002; Langellotto & Denno, 2004; Scherber et al., 2010; Cook-Patton et al., 2011; Chaplin-Kramer & Kremen, 2012), but the potential indirect link between substrate heterogeneity and arthropod diversity, via the plant community, has not been studied.

Several studies have examined factors that may contribute to maximizing the diversity of arthropods specifically on green roofs. The factors that were found to enhance biodiversity were high vegetation cover (Schindler, Griffith & Jones, 2011; Gonsalves, 2016), larger roof size (Berthon, 2015), connectivity with other potential habitats (Berthon, 2015; MacIvor, 2016; Blank et al., 2017; Ksiazek-Mikenas et al., 2018;  Wang et al., 2017), greater vegetation complexity (i.e., height) (Madre et al., 2013; Gonsalves, 2016), plant species diversity (Gonsalves, 2016; Kratschmer, Kriechbaum & Pachinger, 2018; Ksiazek-Mikenas et al., 2018), and greater substrate depth (Kratschmer, Kriechbaum & Pachinger, 2018), or substrate depths under 10 cm or over 15 cm (Kyrö et al., 2018). On the other hand, the type of green roof, extensive or intensive, (Joimel et al., 2018), roof age (Schrader & Böning, 2006), and vegetation cover (Rumble & Gange, 2013) did not affect soil microarthropod diversity in other studies. It was suggested that habitat heterogeneity may be an important factor in increasing these species’ diversity (Rumble & Gange, 2013). However, the direct and indirect effects of the substrate composition and heterogeneity on arthropods have not been examined on green roofs.

Increasing plant diversity can be used as a tool to enhance and maintain diversity at higher trophic levels on green roofs, which can be a source of biodiversity in urban environments. In this study, an attempt was made to enhance plant diversity by increasing substrate heterogeneity and hypothesized indirect positive effects of substrate heterogeneity on arthropod diversity, via a positive effect of substrate heterogeneity on plant diversity. It was hypothesized that substrate heterogeneity would have: (1) a negative effect on herbivore abundance and positive effect on herbivore diversity, as predicted by the enemies hypothesis; (2) a positive effect on predator and parasitoid abundance and diversity; and (3) a positive effect on overall arthropod diversity and abundance. It was also predicted that different levels of substrate heterogeneity will support different arthropod communities due to the different niches each substrate type provided.

## Materials and Methods

### Study sites

The study was conducted on the roofs of three schools in Haifa, Israel, separated by 1.2–2.4 km from each other. Haifa has a Mediterranean climate, with a mean rainfall of 539 mm per year, with most of the rain falling between November and March. Temperatures have a mean minimum of 10 °C and mean maximum of 18 °C in the winter and a mean minimum of 24 °C and mean maximum of 29 °C in the summer. The roofs where the experiment was conducted are referred to here as Dinur School (32.793N, 35.01E), Ben Gurion School (32.79N, 35.0E), and Matos School (32.805N, 34.986E). Dinur School (two floors tall) is surrounded by buildings and limited vegetation, including trees such as *Ailanthus altissima* (Mill.) Swingle, *Dalbergia sissoo* Roxb., and *Ficus spp*. L., Ben Gurion School (three floors tall) is located near a wooded area, with vegetation dominated by *Pinus halepensis* Mill., and Matos School (four floors tall) is located near an ephemeral river channel and a park-like zoo, with woody species such as *Quercus calliprinos* Webb and *Pistacia palaestina* Boiss.

### Experimental design

The experimental design consisted of four treatments varying in substrate heterogeneity. Each treatment was replicated six times, similarly to other studies of effects of soil heterogeneity on arthropods which had 4–12 replicates (Hansen & Coleman, 1998; Kaneko & Salamanca, 1999), with two replicate plots on each of the three roofs. The 24 green roof plots (4 treatments × 3 roofs × 2 replicates per roof) consisted of experimental plots that were 2 × 2 m (4 m^2^) with untreated wooden frames, a 0.5 mm waterproof plastic membrane sheet (Wepelen^®^ Aqua Tec, RKW, Germany), a 2 cm drainage mat composed of recycled polyethelene foam waste (3RFOAM; ‘Palziv’, Ein-Hanatziv, Israel), and substrate composed of 10% peat, 10% compost, 10% tuff (local volcanic ash—0-8 mm) and 70% processed perlite (imported amorphous volcanic glass—0.6 mm, produced by ‘Agrical’, HaBonim, Israel). Plots were spaced at least 0.5 m apart and were separated by bare roof. Heterogeneity of substrate was produced by creating two or four 0.5 × 0.5 m patches of substrate. This is a scale of heterogeneity that has been shown to be relevant to soil microarthropods (Anderson, 1978; Hansen & Coleman, 1998; Kaneko & Salamanca, 1999), and that results in a volume of substrate that would be large enough to contain an ant colony of some species (Tschinkel, 2005). In addition, patch size was sufficient to contain several individuals of the plant species used. The patches contained higher or lower concentration of the various components within a matrix that was similar to the homogenous substrate, but adjusted to achieve a consistent ratio of substrate components across the entire plot, so there would not be a higher percentage of organic matter in the plot as a whole. The treatments included: (1) Homogeneous dispersion (i.e., ‘HOM’)—all components evenly distributed across the 4 m^2^ plot, with no patches; (2) Mineral heterogeneity (i.e., ‘M-HET’)—heterogeneous dispersion of mineral components (tuff and perlite), with two 0.25 m^2^ patches of 80% tuff substrate; (3) Organic heterogeneity (i.e., ‘O-HET’)—heterogeneous dispersion of organic components of substrate (compost and peat), with two patches containing 5% organic matter; (4) Both mineral and organic heterogeneity (i.e., ‘M + O-HET’)—heterogeneous dispersion of both organic and mineral components, with two patches high in tuff and two patches low in organic matter. To maintain the ratio of tuff:perlite and low:high organic matter, the overall composition of the substrate in treatment 4 was 10% peat, 10% compost, 13% tuff, and 67% perlite. Two hundred seeds for each of 20 native annual species (4,000 seeds total, Table 1) were seeded in each plot on December 2nd, 2013, and the plots and seeds were covered with a thin layer of white gravel. As is generally done on extensive green roofs, plots were neither irrigated nor gardened. At each site, rainfall was measured with a rain gauge, and air temperature was measured with a data logger (WatchDog B100; Spectrum Technologies Inc., Aurora, IL, USA) every two hours during the second year of the experiment.

**Table 1:** List of annual species planted in the experimental plots.

Species	Family
*Anthemis pseudocotula* Boiss.	Asteraceae
*Chrysanthemum coronarium* L.	Asteraceae
*Cichorium endivia* L.	Asteraceae
*Hirschfeldia incana* (L.) Lagr.-Foss.	Brassicaceae
*Ricotia lunaria* (L.) DC.	Brassicaceae
*Sinapis alba* L.	Brassicaceae
*Chaetosciadium trichospermum* (L.) Boiss.	Apiaceae
*Daucus broteri* Ten.	Apiaceae
*Tordylium carmeli* (Labill.) Al-Eisawi & Juri	Apiaceae
*Trifolium purpureum* Loisel.	Fabaceae
*Trifolium stellatum* L.	Fabaceae
*Lagurus ovatus* L.	Poaceae
*Stipa capensis* Thunb.	Poaceae
*Echium judaeum* Lacaita	Boraginaceae
*Heliotropium hirsutissimum* Grauer	Boraginaceae
*Agrostemma githago* L.	Caryophyllaceae
*Silene aegyptiaca* (L.) L. f.	Caryophyllaceae
*Lomelosia prolifera* (L.) Greuter & Burdet	Dipsacaceae
*Erodium malacoides* (L.) L’Her.	Geraniaceae
*Malva parviflora* L.	Malvaceae

### Arthropod sampling methods

**Figure 1:**
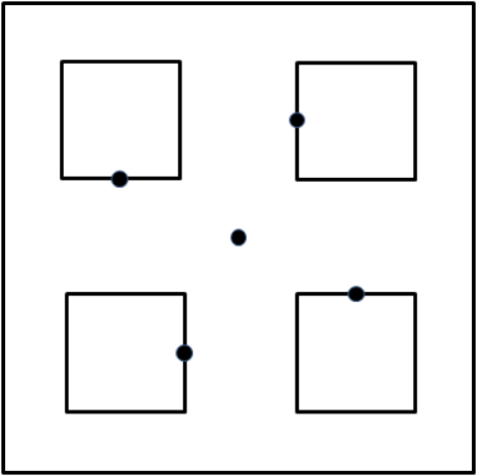
Location of pitfall traps in plots. Illustration of plot with subplots and pitfall traps indicated as dark dot. In the homogenous treatment, all subplots contained the same substrate as the matrix, and in treatments M-HET and O-HET subplots 2 and 3 contained the same substrate as the matrix.

Arthropod abundance and diversity were measured by collecting arthropods in five pitfall traps in each plot during the three years of the study. Traps were placed in the center of the plot and on the borders of the four 0.5 × 0.5 m patches (Fig. 1). Traps were not placed in the center of the subplots in order to avoid reducing the size of the subplots, and also because we expected the species sampled to be sufficiently mobile so that they could also be captured at the subplot edge. For example, a distance of 300 cm is not expected to pose a limit to dispersal of Collembola (Åström & Bengtsson, 2011). The traps consisted of a clear plastic cup, 9 cm in height and 6.5 cm in diameter at the top, containing 20 ml of 80% ethylene glycol, and placed inside another cup that was permanently sunk in the ground with the rim at surface level. Traps were active for two days per month, primarily during the rainy season, in parallel with plant sampling dates. In the first year, traps were active between the months February and April, in the second year between November and May and in July, and in the third year between November and May. When the traps were inactive, the ethylene glycol cup was replaced with a cup filled with gravel. A clear petri dish, 9 cm in diameter, was used as a roof above the traps to reduce filling of traps with rainwater and plant debris. Arthropods were identified to morphospecies (Oliver & Beattie, 1996), with ants also identified to genus or species in order to separately analyze ant species that are closely associated with soil. The Chao index, an index that provides an estimate of true species richness by accounting for the number of collected species that are represented by one or two individuals, was used to estimate the number of species present. Collembola, soil-nesting ants, and mites were analyzed separately, in addition to being included in analyses of the overall community. In addition, arthropod morphospecies were categorized as herbivores, parasitoids, or predators, and each functional group was analyzed separately.

### Vegetation sampling methods

Abundance of plant species was evaluated using point-intercept method (Jonasson, 1988). One hundred skewers, 2.5 mm in diameter, were placed uniformly in each plot, and the number and identity of contacts with green plants were recorded. These data were collected monthly during the growing season for three years (Year 1: Feb–April; Year 2: Nov–July; Year 3: Oct–May). In the first year, since plots were constructed in December and rains began late in the season, sampling began in February. The beginning and end of the sampling was dependent on the timing of rain and sprouting and drying of the vegetation in each year. As described in the statistical analysis section, data from the peak of the season in February or early March were used for some analyses. The point intercept data were used to calculate abundance of plants by summing the total number of contacts of plants with skewers in each plot, and the Shannon–Wiener (H′) index of plant diversity for each plot using the number of contacts of each species with skewers in each plot. In this case H′was used instead of Chao because the precise number of species present was known and only their abundances varied. Also, H′was used in another study at these sites (Vasl, 2016), so it is used here to allow comparison of results.

### Statistical analysis

The effects of substrate heterogeneity on arthropod community composition during years 2 and 3 were examined by non-metric multidimensional scaling (NMDS) visualization and PERMANOVA analysis. Data from the first year were omitted for clarity, as the community was beginning to develop, was drastically different, and would distort the mapping of the data points from other years. Bray–Curtis community similarity values of each plot were mapped on two NMDS axes, and a PERMANOVA was performed on the Bray-Curtis values with treatment and roof as independent factors, for each year separately. The diversity in each plot is indicative of alpha diversity, while the community composition differences across roofs are indicative of beta diversity.

The effects of substrate heterogeneity treatment on overall arthropod abundance and Chao diversity index, and abundance and Chao diversity index of specific arthropod groups (ants, Collembola, mites, and functional groups including herbivores, predators, and parasitoids), were tested with a repeated measures general linear model that included the total diversity or abundance of arthropods per year of the study as a dependent variable, year as the within-subject factor, and treatment, and school roof as between-subject factors. Generally, all three years of the study were used for analyses comparing treatments. In some cases, data from one of the years were not included in the analysis because the abundance or diversity of the arthropod taxon or functional group was uniformly low, and prevented a normal distribution of model residuals, as indicated in the results. Data were ln-transformed as needed to achieve the model requirements. Where Mauchly’s sphericity test indicated that sphericity could not be assumed, a Huynh-Feldt adjustment of degrees of freedom was used.

An indirect effect of the heterogeneity treatment on arthropods, mediated by an effect on plant diversity (H′), was tested. A general linear model was used to test the effect of plant H′as a covariate on the abundance and diversity of arthropod functional groups in February-early March of years 2 and 3, with year included as a random factor and roof as a fixed factor. The effect of plant H′and abundance as a covariate on overall arthropod species richness and abundance was also analyzed with each month’s plant and arthropod abundance or diversity considered as a data point, with date as a random factor and roof as a fixed factor, also in a general linear model. A test was performed for model requirements as described above.

## Results

**Figure 2:**
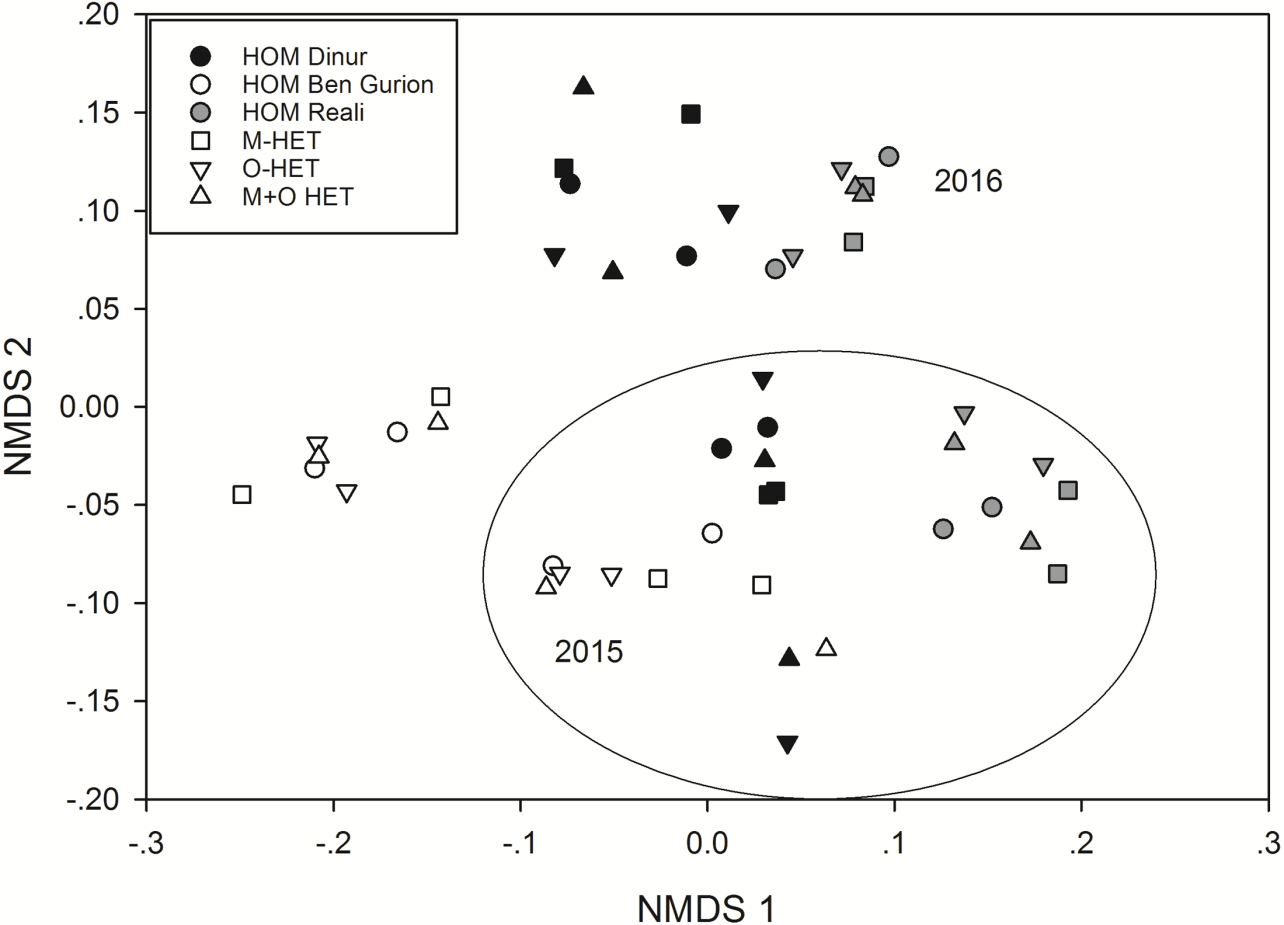
Non-metric multidimensional scaling of Bray-Curtis arthropod community similarity values for last two years of study. Non-metric multidimensional scaling of Bray-Curtis arthropod community similarity values for last two years of study. Shapes of symbols indicate treatment (HOM, homogenous substrate, M-HET, heterogeneous substrate with subplots of tuff, O-HET, heterogeneous substrate with subplots of low organic matter, M + O-HET, heterogeneous substrate with both types of subplots) and color indicates roof. Stress value for mapping of two axes = 0.16.

The overall composition of the arthropod communities was expected to differ based on the substrate types of each treatment, at the alpha diversity level, but found no significant effect of treatment on community composition, while in both years different roofs had different communities (PERMANOVA, P_roof_ = 0.001, Fig. 2), indicative of beta diversity among roofs. In 2015, the MATOS roof had a distinct community from the other roofs, while in 2016 a distinct community was found in the Ben Gurion roof (Fig. 2).

The dominant taxa encountered were Collembola, mites, ants (including *Tetramorium spp.*, *Paratrechina longicornis*, *Monomorium*, and *Pheidole*), aphids, and flies of the family Sciaridae. In the first year, a mean of 250 ± 21 (Mean ± SE) arthropod individuals were collected per plot, in the second year 1,080 ± 48, and in the third year 2,000 ± 196. By the third year, 259 species were collected overall throughout the three roofs, and a mean of 170 ± 3 per roof. Beta diversity, the ratio of diversity across all roofs (gamma diversity) to mean diversity per roof (mean alpha diversity) was 1.53. Thirty five percent of species were found on all roofs, and 40% were found on only one roof. Abundance of arthropods generally peaked around November through December and February through March. Species diversity peaked in March with a mean of 11.6 ± 0.7, 21 ± 1 and 25 ± 0.8 species in the first, second, and third year, respectively.

**Figure 3:**
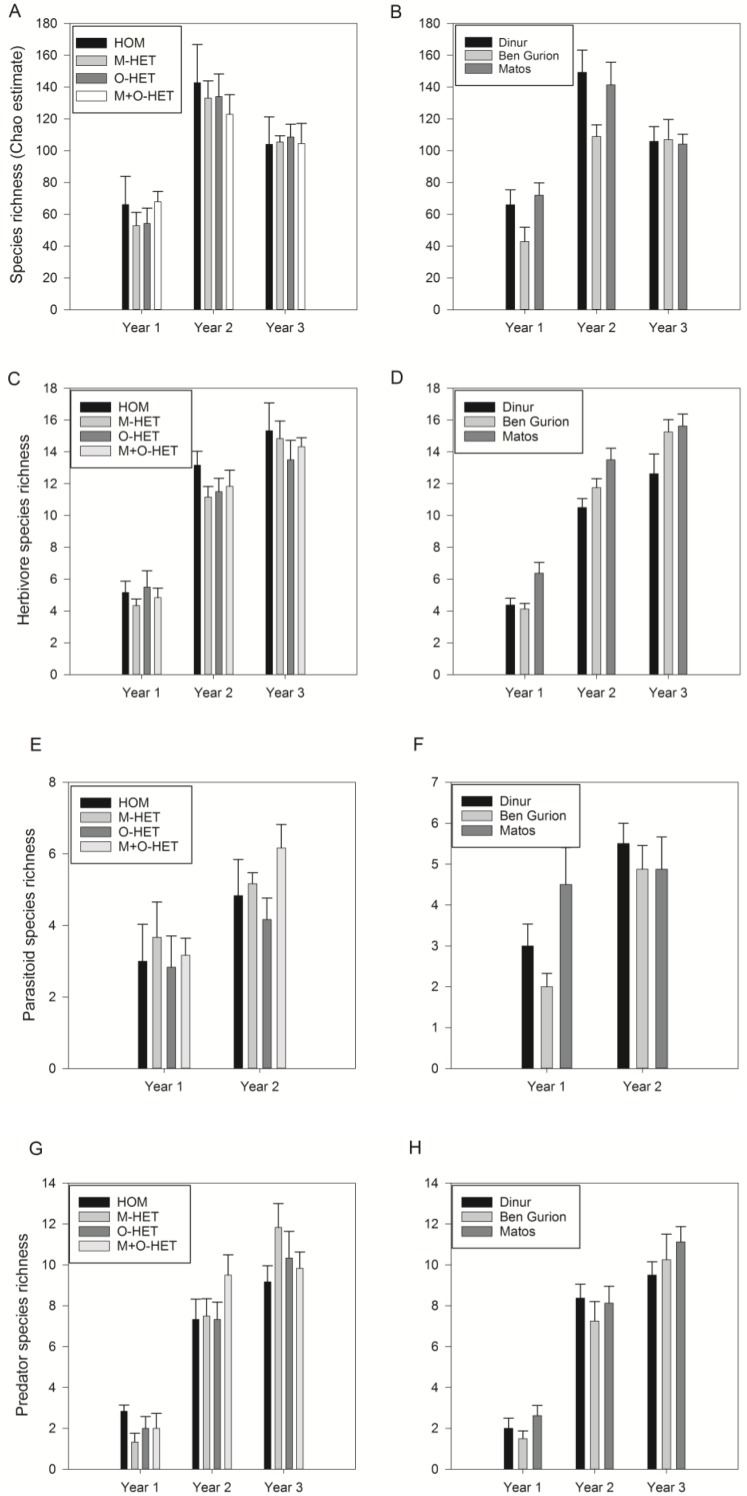
Species richness of arthropod taxa. Species richness of all arthropods (A, B), herbivores (C, D), parasitoids (E, F), and predators (G, H) in pitfall trap samples throughout three years of study. Samples separated by treatment (HOM, homogenous substrate, M-HET, heterogeneous substrate with subplots of tuff, O-HET, heterogeneous substrate with subplots of low organic matter, M + O-HET, heterogeneous substrate with both types of subplots) (A, C, E, G), or separated by roof (B, D, F, H). Bars indicate mean + standard error.

**Figure 4:**
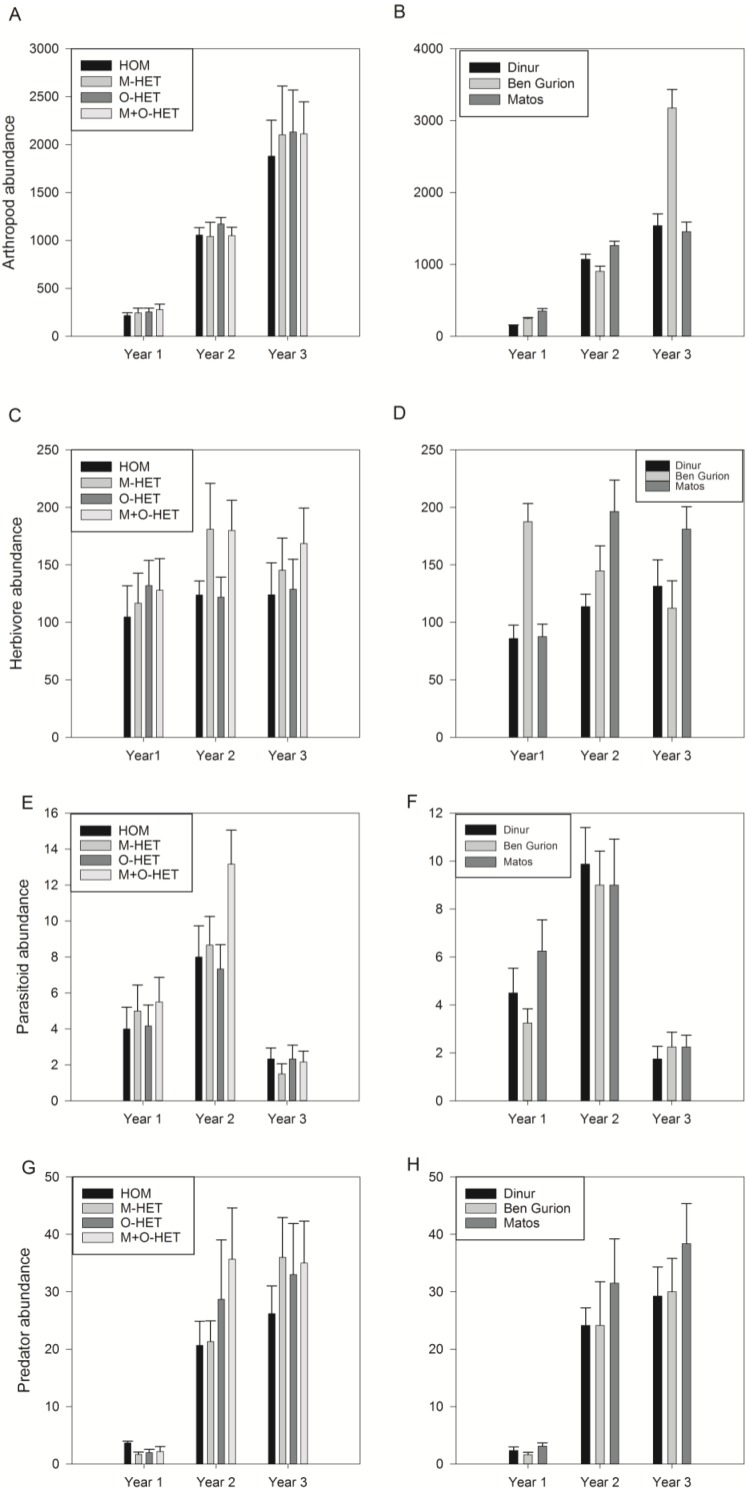
Abundances of arthropod taxa. Abundance of all arthropods (A, B), herbivores (C, D), parasitoids (E, F), and predators (G, H), in pitfall trap samples throughout three years of study. Samples separated by treatment (HOM, homogenous substrate, M-HET, heterogeneous substrate with subplots of tuff, O-HET, heterogeneous substrate with subplots of low organic matter, M + O-HET, heterogeneous substrate with both types of subplots) (A, C, E, G), or separated by roof (B, D, F, H). Bars indicate mean + standard error.

There was no significant effect of treatment on the Chao diversity index (Fig. 3A, Table 2)—diversity at the alpha level, or on overall arthropod abundance (Fig. 4A, Table 3), while there was an effect of roof on overall arthropod richness (Fig. 3B, Table 2)—diversity at the beta level, and abundance (Fig. 4B, Table 3). There was also an interaction between year and roof (Table 3). In the third year twice as many arthropods were collected in Ben Gurion than in the other roofs (Fig. 4B), and in the first and second years Ben Gurion had about 40% fewer species than the other roofs (Fig. 3B). Soil heterogeneity treatment also had no significant effect on species richness and abundance of herbivores, predators, and parasitoids (Figs. 3C, 3E, 3G, 4C, 4E, 4G, Tables 2 and 3). There was, however, an effect of roof on herbivore species richness (Fig. 3D, Table 2). Herbivore species richness was 50% higher in Matos in the first year, and richness was 20% lower in Dinur in the third year (Fig. 3D).

**Table 2:** General linear model results of effects of year, treatment, and roof on species richness of arthropod functional groups and Chao estimate of species richness of all arthropods. Degrees of freedom, *F* values, and *P* values for each of the independent factors in each model are presented. Significant *P* values are represented in bold.

Source of Variation	Herbivore species richness			Parasitoid species richness			Predator species richness			Arthropod species richness (Chao)		
	***df***	***F***	***P***	***df***	***F***	***P***	***df***	***F***	***P***	***df***	***F***	***P***
Treatment	3	0.80	0.520	3	0.73	0.554	3	0.24	0.868	3	0.29	0.829
Roof	2	7.05	**0.009**	2	1.44	0.274	2	0.82	0.463	2	5.44	**0.021**
Treatment*roof	6	0.47	0.821	6	0.53	0.777	6	0.57	0.746	6	0.54	0.771
Year	2	139.36	**<0.001**	1	17.93	**0.001**	2	113.75	**<0.001**	2	33.91	**<0.001**
Year*treatment	6	0.59	0.736	3	0.69	0.576	6	1.97	0.110	6	0.32	0.921
Year*roof	4	1.06	0.396	2	2.96	0.090	4	0.64	0.635	4	1.20	0.336
Year*treatment*roof	12	0.79	0.659	6	1.61	0.226	12	0.97	0.503	12	1.72	0.125
Error	24			12			24			24		

**Figure 5:**
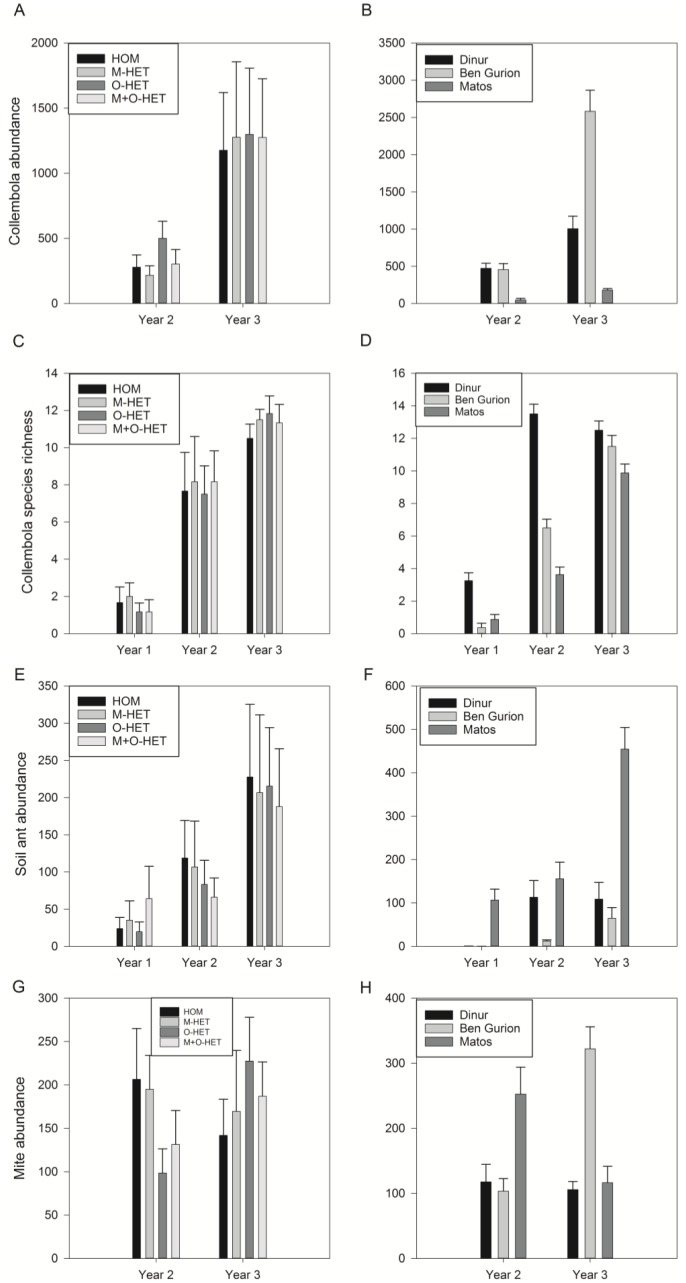
Abundance or species richness of soil arthropods. Abundance or species richness of collembola (A, B, C, D), soil-nesting ants (E, F), and mites (G, H) in pitfall trap samples throughout two or three years of study. Samples separated by treatment (HOM, homogenous substrate, M-HET, heterogeneous substrate with subplots of tuff, O-HET, heterogeneous substrate with subplots of low organic matter, M + O-HET, heterogeneous substrate with both types of subplots) (A, C, E, G), or separated by roof (B, D, F, H). Bars indicate mean + standard error.

The abundance of arthropods associated with the soil, including Collembola, soil-nesting ants, and mites, were not significantly affected by heterogeneity treatment, while there was an effect of roof, and an interaction between roof and year (Fig. 5, Table 4). Collembola abundance was ten times higher in Dinur and Ben Gurion than in Matos in the second year, and 5.5 times higher in Dinur and 15 times higher in Ben Gurion than in Matos in the third year. The species richness of Collembola was also not affected by treatment (Fig. 5, Table 4).

**Figure 6:**
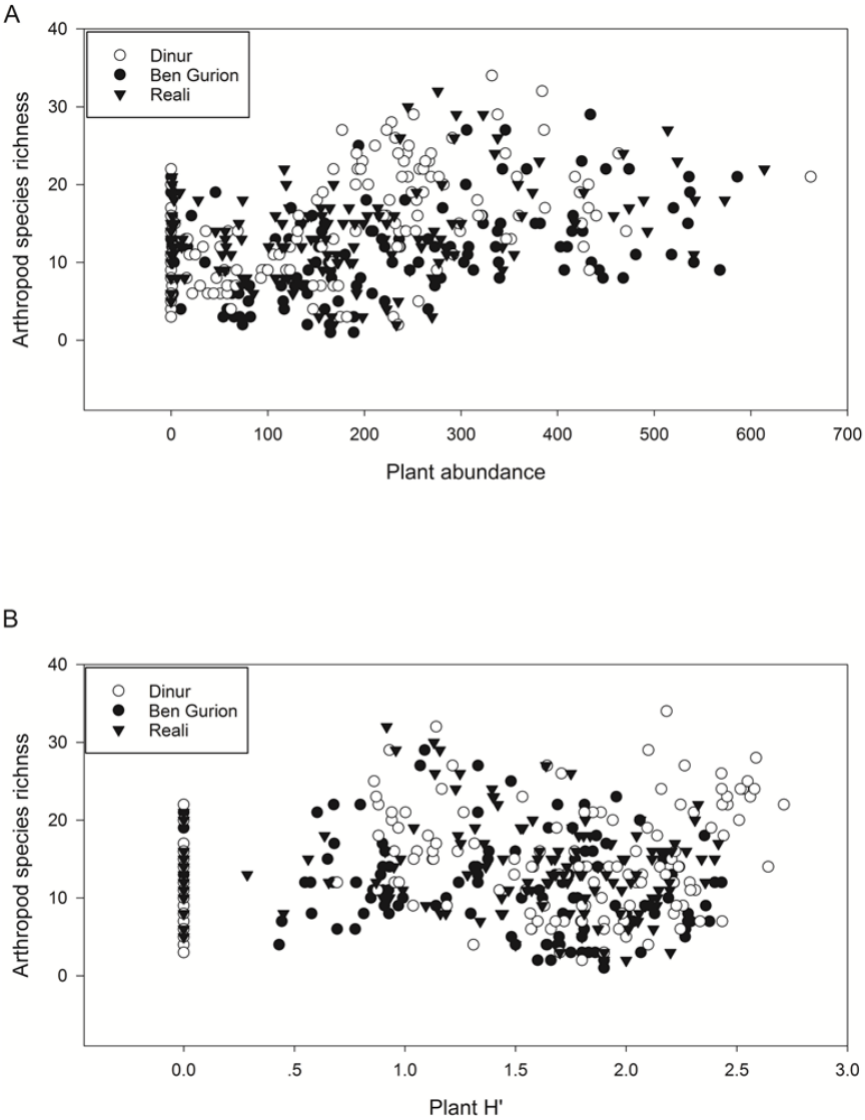
Correlation of arthropod species richness with plant variables. Correlation of arthropod species richness with A. plant abundance, measured as total touches of plants with skewers in point intercept measurement, and B. plant Shannon-Weiner diversity index (H′). Each datapoint represents a plot in a given month throughout the experiment, which was included in a general linear model with date as a random factor and roof as a fixed factor and plant abundance or diversity as covariates. Different roofs are indicated by different symbols.

There was no significant effect of heterogeneity on plant diversity and biomass on the whole plot scale (Vasl, 2016), though there were some differences at the subplot level. Namely, in the M-HET treatment plant biomass was higher in the subplots than in the matrix, and the M-HET and O-HET subplots’ plant communities were different from plant communities in other locations during the first year (Vasl, 2016). However, since there was no effect overall at the whole plot scale, there was no clear indirect effect of substrate heterogeneity on arthropod diversity via an effect on the plant community. Plant abundance (but not plant H′) had a significant positive effect on arthropod diversity when included as covariates in a general linear model (Fig. 6, Table 5). The abundance of functional groups, herbivores and predators and parasitoids, was not affected by plant H′(Table 6). Mean herbivore abundance in the peak of the season in February–March was 39 ± 4, and mean predator and parasitoid abundance was 10 ± 1 individuals. Herbivore diversity and predator and parasitoid diversity were also not affected by plant H′(Table 6). Mean herbivore diversity in February–March was 3.5 ± 0.2 species, and mean predator and parasitoid diversity was 3.5 ± 0.3 species.

**Table 3:** General linear model results of effects of year, treatment, and roof on abundance of arthropod functional groups and all arthropods. Significant *P* values are represented in bold.

Source of Variation		Herbivore abundance		Parasitoid abundance		Predator abundance		Arthropod abundance	
	***df***	***F***	***P***	***F***	***P***	***F***	***P***	***F***	***P***
Treatment	3	1.71	0.219	1.29	0.323	0.47	0.710	0.40	0.758
Roof	2	3.75	0.054	0.37	0.699	0.71	0.510	12.48	**0.001**
Treatment*roof	6	0.23	0.958	0.44	0.836	0.71	0.650	0.46	0.827
Year	2	2.66	0.091	32.60	**<0.001**	42.92	**<0.001**	125.63	**<0.001**
Year*treatment	6	0.78	0.592	1.26	0.312	0.87	0.533	0.17	0.982
Year*roof	4	8.02	**<0.001**	0.76	0.564	0.32	0.864	19.24	**<0.001**
Year*treatment*roof	12	1.76	0.116	0.53	0.876	1.47	0.204	0.46	0.918
Error	24								

There was some variation in local meteorological conditions between roofs, with Matos receiving about 50 mm more rain than the other schools during the second year, and temperatures were 2–4 °C higher in Dinur School than the other schools, likely because the roof was darker (Vasl, 2016). However, yearly rainfall and maximum temperature of each roof were not correlated with arthropod species richness and abundance of the roof (Fig. S1).

## Discussion

The relationship between habitat heterogeneity and species diversity is generally predicted to be positive (Stein, Gerstner & Kreft, 2014). In particular, substrate heterogeneity was expected to enhance plant diversity, and via this effect on plant diversity, enhance arthropod diversity. However, there was no effect of plant diversity on arthropod diversity, perhaps because substrate heterogeneity did not affect the plant community (Vasl, 2016). Though there were some effects at the subplot level, there was no lasting overall effect of heterogeneity treatment at the entire plot level.

Thus, there could be no effect of substrate heterogeneity on arthropods at the level of the whole community via the effect on vegetation diversity and abundance. Nevertheless, when testing for an effect of vegetation on arthropods directly, plant abundance did affect the arthropod communities, as expected, but plant diversity did not affect the arthropod communities. This is in contrast to previous studies, which indicated that there is a positive correlation between plant diversity and arthropod diversity, and only a weak effect of plant abundance on arthropod diversity (Borer, Seabloom & Tilman, 2012), or that showed no effect of an increase in plant abundance, due to fertilization, on arthropod abundance or diversity, except of ground dwelling herbivores and detritivores (Asmus et al., 2017). There have also been studies specifically on green roofs that indicated an effect of plant diversity on arthropod diversity (Ksiazek-Mikenas et al., 2018). They also noted an effect of roof size on spider diversity, which may have be indicative of an effect of heterogeneity (Ksiazek-Mikenas et al., 2018), but not necessarily substrate heterogeneity, and could also be the result of greater colonization rates on larger roofs. The lack of a plant diversity effect in the current study is most likely because there was no difference in the number of plant species, which was generally identical among plots, and differences were only in the relative abundances of different species.

**Table 4:** General linear model results of effects of year, treatment, and roof on abundance or species richness of substrate-associated arthropods. Significant *P* values are represented in bold.

Source of Variation	Collembola abundance			Collembola species richness			Soil ant abundance			Mite abundance		
	***df***	***F***	***P***	***df***	***F***	***P***	***df***	***F***	***P***	**df**	***F***	***P***
Treatment	3	0.32	0.809	3	0.55	0.657	3	0.11	0.950	3	0.22	0.878
Roof	2	35.82	**<0.001**	2	75.85	**<0.001**	2	26.45	**<0.001**	2	7.32	**0.008**
Treatment*roof	6	0.21	0.97	6	1.22	0.359	6	0.75	0.624	6	0.47	0.818
Year	2	62.41	**<0.001**	2	364.78	**<0.001**	2	22.52	**<0.001**	1	6.63	0.369
Year*treatment	6	0.19	0.967	6	0.76	0.604	6	0.37	0.892	3	0.87	0.078
Year*roof	4	21.76	**<0.001**	4	25.01	**<0.001**	4	8.29	**<0.001**	2	16.92	**<0.001**
Year*treatment*roof	12	0.26	0.990	12	2.09	0.060	12	0.46	0.917	6	0.30	0.925
Error	24			24			24			12		

In addition, a direct effect of substrate heterogeneity on arthropods would be expected, but there was no effect on abundance and/or diversity of groups that are associated with the substrate, including Collembola, some ant species, and mites, although the substrate was clearly heterogeneous throughout the experiment (Vasl, 2016). Mites and Collembola, for example, though affected by heterogeneity at a scale of millimeters to tens of centimeters, may be more affected by the differences in types of organic materials in the substrate (Anderson, 1978; Hansen & Coleman, 1998; Kaneko & Salamanca, 1999). Also, another study has already shown a case where soil microarthropods were not affected by substrate characteristics such as organic and water content (Gutiérrez-López et al., 2010). In the current study, heterogeneity was in quantity, rather than type of organic matter, which appeared to have no effect on mites and Collembola, so future studies focusing on these taxa may examine a greater range of variation in substrate types. In general, substrate heterogeneity is expected to contribute to soil arthropod diversity, but most studies showed an effect on diversity of certain taxa within patches, but did not test for an overall effect in the area encompassing the patches (Decaëns, 2010). In this study, where diversity was examined at a scale that encompasses the heterogeneity of patches and matrix, no effect was found.

## Conclusions

While heterogeneity treatments did not affect the arthropod communities, there was an effect of roof on the arthropod community, with Collembola, soil ant, and mite abundance, overall arthropod abundance, richness of Collembola, herbivores, and all arthropods, and overall community composition, differing among roofs. This suggests that external factors in the area surrounding the roof had a greater effect than the treatment imposed within the experiment. Previous studies have shown similar effects of the surrounding community on green roof fungal (McGuire et al., 2013) and arthropod (Braaker et al., 2014) communities, as green roof communities were similar to nearby green roofs or ground-level habitats. As seen by comparing alpha and gamma diversity, diversity across roofs was greater than the diversity on each roof, and there was species turnover among roofs. These results point to a factor that could be used to protect biodiversity on green roofs in cities; by building green roofs in different areas of the city, surrounded by different habitat types, the green roofs can serve as an extension of these habitats and further support the arthropod communities found there. Thus, a diversity of arthropod communities may be protected even if the plant communities are similar.

**Table 5:** General linear model results of effects of date (every month of every year of data collection), roof, and plant abundance and plant H′as covariates on arthropod species richness. Significant *P* values are represented in bold.

Source of Variation	Plant abundance as covariate			Plant H′as covariate		
	***df***	***F***	***P***	***df***	***F***	***P***
Plant abundance/H′	1	7.18	**0.008**	1	0.96	0.329
Roof	2	6.06	**0.006**	2	4.78	**0.015**
Date	17	7.99	**<0.001**	17	10.56	**<0.001**
Date*roof	34	5.95	**<0.001**	34	5.80	**<0.001**
Error	377			377		

**Table 6:** General linear model results of effects of year, roof, and plant Shannon diversity index as covariate on arthropod functional group abundance and diversity. Significant *P* values are represented in bold.

Source of Variation	Herbivore abundance			Predator and parasitoid abundance			Herbivore diversity			Predator and parasitoid diversity		
	***df***	***F***	***P***	***df***	***F***	***P***	***df***	***F***	***P***	***df***	***F***	***P***
Plant H′	1	2.15	0.151	1	0.18	0.675	1	3.99	0.053	1	0.002	0.961
Roof	2	1.77	0.351	2	3.12	0.069	2	1.76	0.355	2	0.649	0.586
Year	1	0.21	0.667	1	0.46	0.501	1	3.50	0.129	1	0.073	0.789
Year*roof	2	4.25	**0.021**	2	0.07	0.937	2	5.35	**0.009**	2	0.790	0.461
Error	41			41			41			41		

##  Supplemental Information

10.7717/peerj.6445/supp-1 Data S1 Experimental Data

10.7717/peerj.6445/supp-2 Figure S1 Correlation of number of arthropod species on each roof in each year with climatic conditions (A) Maximum temperature on roof in January. Pearson correlation results: *r*_2015_ = 0.049, *P* = 0.969. *r*_2016_ =  − 0.602, *P* = 0.589. (B) Precipitation on roof in each year. Pearson correlation results: *r*_2015_ = 0.526, *P* = 0.647. *r*_2016_ =  − 0.676, *P* = 0.528.

## Additional Information and Declarations

### Data Availability

The following information was supplied regarding data availability: The raw data is available as a Supplementary File.
