# Supplementary figures and images for: Fine-scale substrate heterogeneity does not affect arthropod communities on green roofs
Source: PeerJ. 2019 Mar 19;7:e6445. doi: 10.7717/peerj.6445 (PMC6430103; doi:10.7717/peerj.6445)

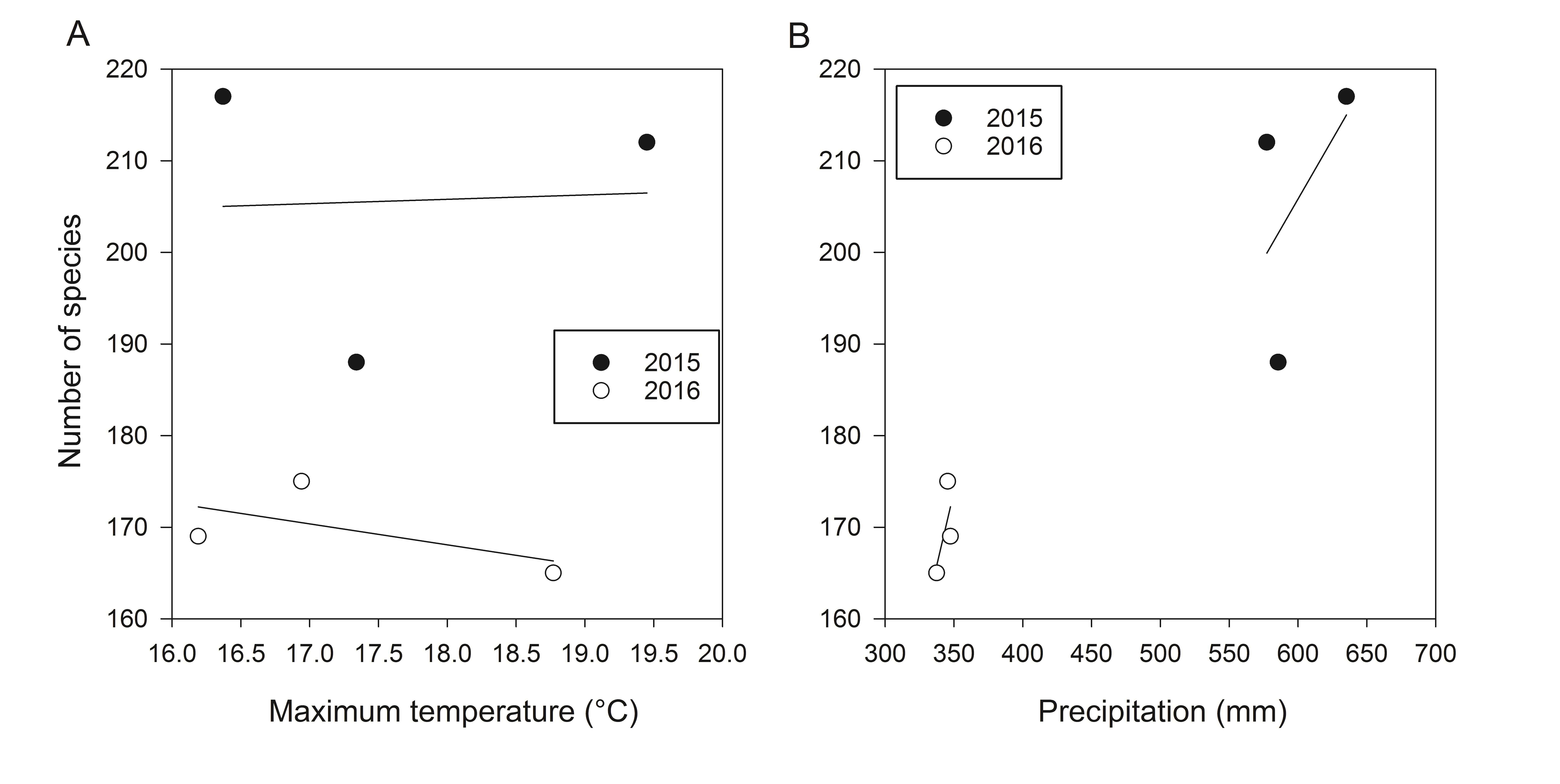

Supplement: Figure S1 — (A) Maximum temperature on roof in January. Pearson correlation results: r2015 = 0.049, P = 0.969. r2016 = − 0.602, P = 0.589. (B) Precipitation on roof in each year. Pearson correlation results: r2015 = 0.526, P = 0.647. r2016 = − 0.676, P = 0.528. [file peerj-07-6445-s002.png]
